# Situating defaunation in an operational framework to advance biodiversity conservation

**DOI:** 10.1093/biosci/biad079

**Published:** 2023-09-19

**Authors:** John R Poulsen, Vincent Maicher, Halina Malinowski, Camille DeSisto

**Affiliations:** The Nature Conservancy, Boulder, Colorado, United States; Duke University, Durham, North Carolina, United States; CAFI Forest Research and Monitoring for The Nature Conservancy, Gabon; Nicholas School of the Environment, Duke University, United States; Nicholas School of the Environment, Duke University, United States

**Keywords:** defaunation, faunal degradation, extinction, extirpation, ecological erosion

## Abstract

Anthropogenic pressures are causing the widespread loss of wildlife species and populations, with adverse consequences for ecosystem functioning. This phenomenon has been widely but inconsistently referred to as *defaunation*. A cohesive, quantitative framework for defining and evaluating defaunation is necessary for advancing biodiversity conservation. Likening defaunation to deforestation, we propose an operational framework for defaunation that defines it and related terms, situates defaunation relative to intact communities and faunal degradation, and encourages quantitative, ecologically reasonable, and equitable measurements. We distinguish between *defaunation*, the conversion of an ecosystem from having wild animals to not having wild animals, and *faunal degradation*, the process of losing animals or species from an animal community. The quantification of context-relevant defaunation boundaries or baselines is necessary to compare faunal communities over space and time. Situating a faunal community on the degradation curve can promote Global Biodiversity Framework targets, advancing the 2050 Vision for Biodiversity.

Since the expansion of humans out of Africa, anthropogenic activities have provoked unprecedented rates of animal extinctions and population declines globally (Cowie et al. 2022). At 100 to 1000 times greater than background rates (Pimm et al. 2014, Ceballos et al. 2015), modern extinction rates greatly surpass those of the past and are accumulating as the sixth mass extinction event (Barnosky et al. 2011, Ceballos et al. 2020). Wild animal populations are declining on land and in the sea (WWF 2020), suggesting additional extinctions are on the horizon. With only 3% of the Earth's land surface considered faunally intact (Plumptre et al. 2021), the global biomass of wild animals is now less than 25% of that before the Pleistocene megafaunal extinction (Bar-On et al. 2018).

The loss of species and populations of wildlife, as well as local declines in the abundance of individuals, is widely known as *defaunation* (for definitions of key terms, see box 1; Dirzo et al. 2014). The concept, first articulated by Dirzo and Miranda in 1991 (Dirzo and Miranda 1991), shares similarities with the empty forest syndrome proposed by Redford (1992) but is more expansive in scope. Defaunation includes four components. First, *wildlife* refers to all living animals that are not humans or domesticated. Therefore, in contrast to American vernacular in which wildlife often denotes vertebrates, defaunation should ideally include other animals, such as arthropods (Dirzo et al. 2014). Second, defaunation incorporates the loss of populations and declines in abundance, which means that extinction and extirpation may be invoked but not necessarily in all circumstances. Third, defaunation can be driven by multiple causes. Whereas the empty forest syndrome stems from overhunting, defaunation recognizes additional drivers such as habitat loss and degradation, wildfire, wildlife parasites or pathogens, human–wildlife conflict, roadkill, mesopredator release and proliferation of invasive species (Dirzo et al. 2014, Young et al. 2016). Fourth, *defaunation* is intended to be analogous to *deforestation*; not only are the terms parallel in structure, with the same prefix (*de-*, meaning the “opposite of” or “removing something”), but in their seminal review, Dirzo and colleagues (2014) wrote that *defaunation* “needs to be considered in the same sense as *deforestation*.”

Box 1. **Glossary.**

*Biotic homogenization* is increased similarity in the composition of species among ecological communities, also referred to as reduced β diversity.

*Body size downgrading* is the loss of large-body animals from ecosystems resulting in smaller average body size.

*Conservation translocation* is the intentional movement of animals to restore populations.

*Defaunation*, defined by Dirzo and colleagues (2014), is the loss of species and populations of animals, as well as local declines in abundance of individuals.

*Defaunation*, defined in the present article, is the conversion of an ecosystem from having wild animals to not having wild animals.

*Deforestation* is the conversion of forested land to nonforested land.

*Ecological erosion* is the incremental deterioration or alteration of ecological processes.

*Empty forest* refers to an ecosystem that is void of large mammals, in which the forest has not been degraded but large mammals are absent as a result of hunting.

*Extinction* is the global loss of a species.

*Extirpation* is the local or regional elimination of a species.

*Faunal conversion* is a change in the composition of a faunal community in which extirpated species are replaced by other species.

*Faunal degradation* is the process of losing animals or species from an animal community.

*Forest degradation* is a process of change within the forest that negatively affects the structure or function of the forest stand or site. In the climate change literature, it is defined as the loss of carbon from forests that remain forests (UNFCCC).

*Trophic downgrading* is the loss of upper trophic position consumers from a community.

Defaunation has triggered an active and rich field of research, with a 90% increase in publications since the empty forest syndrome was first proposed in 1992. All indications are that this phenomenon and line of research will accelerate as declines in species, populations, and abundances of animals are expected to increase with human-caused climate change and land-use change (Pereira et al. 2010). The problem is that *defaunation* is often used simply to describe declines in animals, particularly mammals (but see Hallmann et al. 2017, Fuzessy et al. 2021, Pacoureau et al. 2021), without specification of the degree of animal depletion in numbers or species required for defaunation (e.g., Benítez-López et al. 2017). As an illustration, a Scopus search of the word *defaunation* for the year 2021 retrieved 59 articles in English (supplemental table S1) of which 9 papers were either on an unrelated topic or only anecdotally used the term. Of the 50 remaining papers, 24% used the term ambiguously or indeterminately referred to both declines in animal abundance and species extinction. Forty-four percent used the term to refer to local or regional extirpation of selected groups of organisms, whereas 32% referred to declines in local or regional assemblages (table S1). Most of these studies were focused on terrestrial vertebrates (88%) and, among them, on primary consumers (44%). As such, *defaunation* lacks a universally understood meaning that renders it difficult to compare across space or time.

We contend that the concept of defaunation would be strengthened and made unambiguous if it were used consonantly with deforestation, which it currently is not. We propose a new, quantitative framework for evaluating defaunation that draws on nature-based climate change policy and that aligns with the Kunming–Montreal Global Biodiversity Framework (GBF), agreed at the 15th meeting of the Conference of Parties to the UN Convention on Biological Diversity. In our framework, defaunation, like deforestation, is binary, with accepted reference and endpoints. *Faunal degradation*, similar to *forest degradation*, is the process of losing animals or species from a community. The goal of this framework is to clarify the defaunation concept and associated terms so they are universally and consistently understood, to situate defaunation relative to less severe species and animal losses and alternative endpoints, and to encourage quantitative measures of defaunation that are ecologically reasonable, equitable, and politically expedient, so they can advance biodiversity conservation to meet the 2050 Vision for Biodiversity.

## Defaunation as an analogue to deforestation

Biodiversity loss and climate change are arguably the two greatest environmental threats of this century. Over the past decade, the development of nature-based climate solutions, such as the Reducing Deforestation and Forest Degradation (REDD+) policy framework, to increase carbon storage or avoid greenhouse gas emissions has spurred advances in forest monitoring. In order to finance nations or projects for reducing emissions, concepts such as deforestation and forest degradation needed to be universally understood so that changes in forest carbon could be measured, monitored, and verified (MRV; Putz and Redford 2010). This necessarily led to the development of reference emission levels (REL)—benchmarks of the amount of emissions for a reference period against which future emissions and removals can be compared. Because REDD+ payments are performance based, emission reductions, RELs, and MRV form the backbone of the framework. Emission reductions are measured as verified emissions subtracted from the baseline rate of emissions. There is still not full scientific consensus on global carbon accounting (i.e., Tropek et al. 2014), but enormous progress has been made (Goetz et al. 2015). REDD+ has advanced MRV but has also been strongly criticized for failing to protect rights of local communities and for paying the world's poorer countries to absorb pollution from the world's richest countries (Bayrak and Marafa 2016).

**Figure 1.**
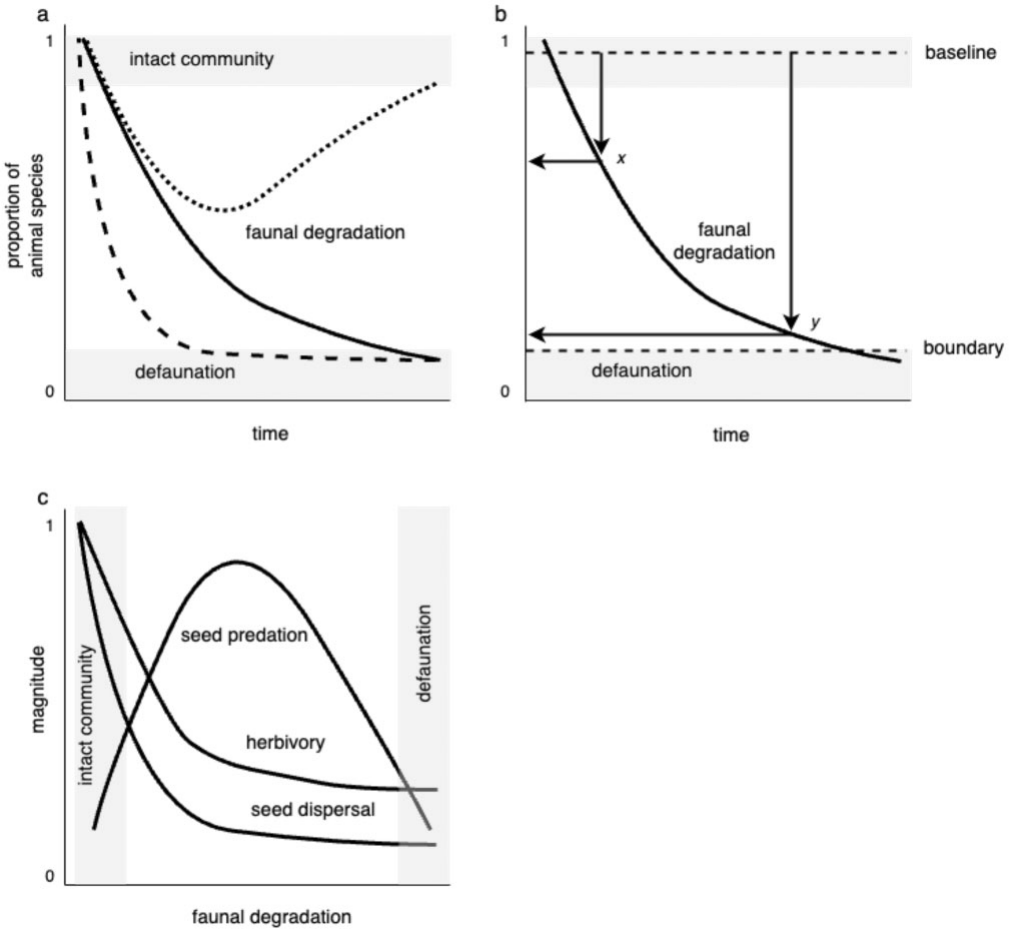
Defaunation conceptual framework. (a) Defaunation is the state where the animal community no longer functionally exists. There are an infinite number of paths an intact community can take toward defaunation. The lines represent different scenarios of faunal degradation. The dashed line depicts a community that is rapidly converted to defaunation, perhaps by habitat conversion. The black line depicts a community that is slowly degraded over time, perhaps by hunting. The dotted line depicts either faunal conversion, where species from the original community are replaced by invasives or species with expanded ranges or successful conservation in which the original community rebounds. (b) The level of faunal degradation can be quantified as multiple attributes of species (dissimilarity, biomass, abundance, density, occurrence, occupancy, functional distinctness). In the present figure, we represent it simply as the difference in proportion of species. Such defaunation indices could be compared across space and time. In this example, the degradation index has decreased by roughly 45% from ***x*** to ***y*** and now lies close to the defaunation boundary. (c) Defaunation and faunal degradation affect ecological processes such as seed predation, herbivory, and seed dispersal. The magnitude or strength of these processes changes with different levels of animal depletion.

In the present article, we borrow from nature-based climate solutions to propose an operational framework of defaunation that is analogous to deforestation (*sensu* Dirzo et al. 2014). *Deforestation* is defined by the Merriam-Webster dictionary as “the action or process of clearing of forests; also, the state of having been cleared of forests” (Merriam-Webster 2023). In the Marrakesh Accords, *deforestation* is defined as “the direct human induced conversion of forested land to nonforested land” (UNFCCC 2001). The Food and Agriculture Organization of the United Nations (FAO) defines *deforestation* as “the conversion of forest to another land use or the long-term reduction of tree canopy cover below the 10% threshold” (FAO 2000). Although organizations define the term slightly differently, at its core, *deforestation* refers to the conversion of forested land to nonforested land. It is a state transition that can be portrayed as a binary variable (forest, nonforest). This process frequently occurs rapidly through swidden agriculture, clearcut logging, clearing for ranching, and wildfire (figure 1a). Moreover, the intent of these activities is often but not always conversion from forest to nonforest.

Therefore, to be analogous to *deforestation*, the simplest definition of *defaunation* would be the transition of an ecosystem from having wild animals to not having wild animals. As such, an ecosystem without wild animals would be *defaunated*, just as a once forested area without trees is *deforested*. And like *deforestation, defaunation* would be recognized as a state transition with an analogous end point. Note, however, that just as *deforestation* does not mean all trees are absent, *defaunation* can occur without the total absence of animals (see the boundaries below). This shift from faunated to defaunated does occur. Whereas extinction often proceeds slowly (Brook et al. 2008), abundance declines can occur rapidly via habitat modification, deforestation, wildfire, and as a result of losing a first species that triggers an extinction cascade (Borrvall et al. 2000).

In contrast to *deforestation, forest degradation* describes the incremental simplifying of forest structure. It stems from selective logging, harvesting for firewood and charcoal, overgrazing, and many other anthropogenic activities (Hosonuma et al. 2012). Forest degradation does not follow a single path; it occurs on continua and follows trajectories affected by thresholds and other nonlinearities (Putz and Redford 2010). But forest degradation consistently negatively affects the structure or function of the forest stand or site, lowering its capacity to supply products and services (Vasquez-Grandon et al. 2018).

*Faunal degradation*, akin to *forest degradation*, is the process by which most ecosystems lose their animals. Typically, animal depletion is a slow and unintended process; animal species and populations are not all lost at once, and several drivers may act synergistically. At local scales, faunal degradation often does not decrease species richness (Finderup Nielsen et al. 2019), whereas at regional scales, biotic homogenization reduces beta and gamma diversity of animals in terrestrial and aquatic settings (Fraser et al. 2022). Faunal degradation can also take multiple, nonlinear pathways. As such, degraded faunal communities lie along a continuum of ecological impoverishment (figure 1a). For example, road construction and habitat fragmentation can degrade the faunal community over time by opening accessibility to humans, exacerbating hunting, exploitation, and habitat destruction. Faunal degradation has been described as a three-phase process proceeding from harvest for direct consumption to commercial exploitation to use of an animal communities’ ecological space (McCauley et al. 2015). This process is often characterized by body size (Smith et al. 2018) or trophic downgrading (Estes et al. 2011) by which larger-body animals, particularly apex predators, are the first to be extirpated followed by smaller animals or those lower on the food chain.

As animal species are lost or reduced in abundance, the processes to which they contribute, such as predation, seed dispersal, herbivory, pollination, decomposition, and nutrient cycling, are weakened and eroded; this is *ecological erosion* (*sensu* Poulsen et al. 2013). At the community level, however, the strength of different processes can change nonlinearly as the faunal community composition changes (figure 1c). In tropical forest systems, the removal of predators, for example, can release consumers from top-down regulation, increasing the populations of herbivores and rodents and intensifying herbivory and seed predation (Terborgh et al. 2001, Williams et al. 2021). In benthic systems, predator removal can increase mesopredator populations’ intensifying pressure on lower trophic levels and exacerbating ecosystem degradation (Myers et al. 2007). As a result of faunal degradation, ecosystem functions and services can directly be affected through species extirpation or indirectly through cascading effects (Bogoni et al. 2020).

**Figure 2.**
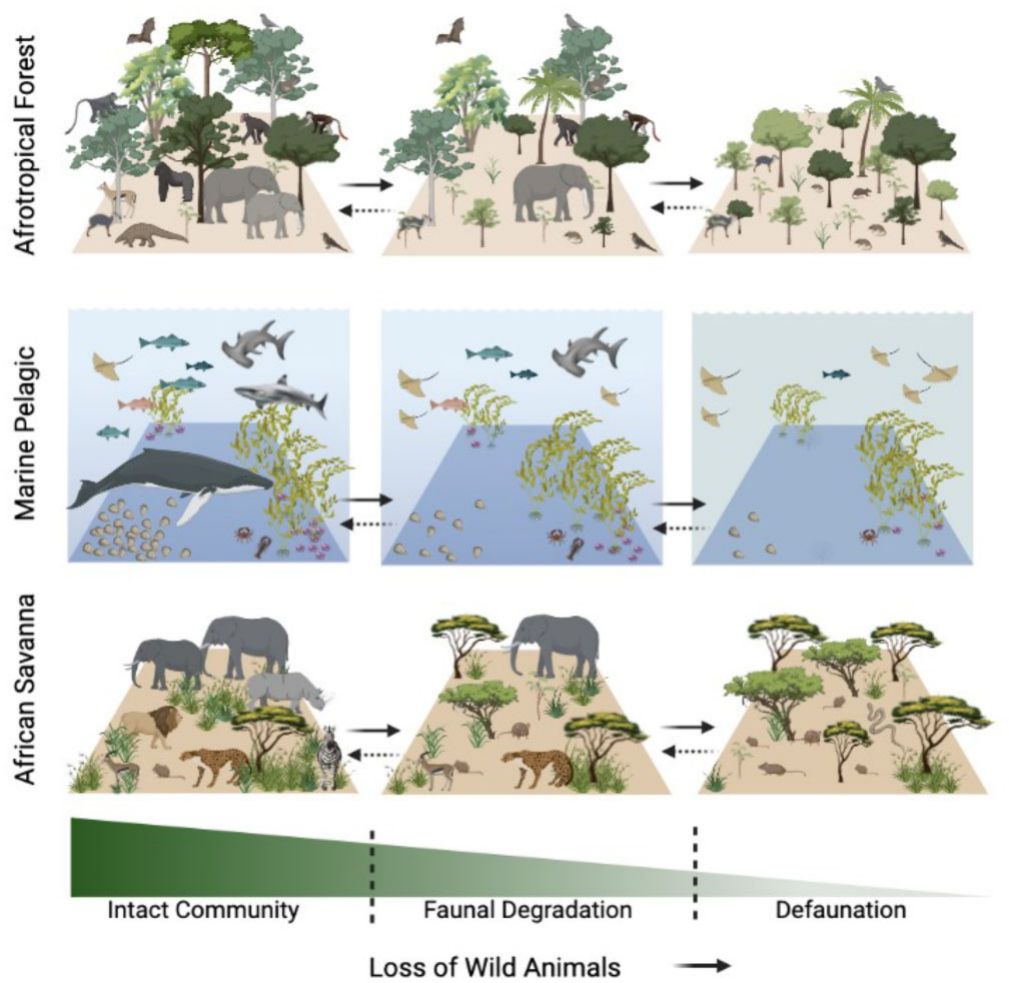
Schematic diagram of intact, degraded, and defaunated communities from Afrotropical rainforest, marine pelagic, and African savanna ecosystems along a defaunation gradient. These are example communities that represent the defaunation process. Faunal degradation and defaunation occur in communities worldwide, with heterogenous paths and consequences for ecosystem function and structure. In most scenarios, this process disproportionately targets large-body animals and results in an increase in abundance of small-body animals released from resource competition and predation. Some large-body species can persist in human-modified landscapes because of their large home range (Poulsen et al. 2011); therefore, it is possible for a community to be defaunated despite the occurrence of large-body species. In addition to the loss of species biodiversity and changes in vegetation biomass, as can be seen above, defaunation can also lead to more nuanced environmental changes such as decreases in nutrient translocation and ecosystem productivity. As was described in the text, defaunation is not necessarily the complete absence of animals. Crossing boundaries between intact communities, faunal degradation, and defaunation is possible and bidirectional. Source: Figure created in BioRender with additional clipart from pinclipart.com, imgbin.com, clipartmax.com, dimensions.com, creazilla.com.

The distinction between defaunation and faunal degradation is important, because it reveals information about the status of the faunal community, the process of animal depletion, and the strength of ecological processes that maintain ecosystem functions and services (figure 2). Just as a degraded forest could regenerate, human-induced faunal degradation does not always result in defaunation, with ecosystems becoming less diverse and wildlife less abundant (Young et al. 2016). Two alternatives exist. First, reduction of human pressures, such as hunting and resource extraction, could allow the community to return to its original state—the goal of many conservation efforts. Second, human activities could permanently change the community composition—faunal conversion—by facilitating species invasion, promoting species naturalization, and altering species ranges. Therefore, faunal degradation might lead to novel ecosystems that are ecologically stable and that provide ecosystem services of their own (Morse et al. 2014).

## Measurement, boundaries, and baselines

The magnitude of defaunation and faunal degradation can be quantified using multiple measures. Giacomini and Galetti (2013) proposed the Bray–Curtis index to measure defaunation by quantifying dissimilarity between a focal and a reference assemblage. This index can be used with abundance and density data, occurrence data, and occupancy probabilities that account for imperfect detection of animals (Giacomini and Galetti 2013). It can also be applied to both species and functional groups of animals (Bogoni et al. 2018). Other studies have compared abundance estimates in hunted areas with those in nonhunted areas and those in disturbed with those in undisturbed areas (Poulsen et al. 2011, Benítez-López et al. 2019). Population viability analysis (Chaudhary and Oli 2020) and early warning signals (Cerini et al. 2023) have also been used to understand extinction risk and to forecast future scenarios of population growth and decline.

To clearly distinguish defaunation from faunal degradation, we must define boundaries. A *boundary* is the magnitude of animal depletion at which an area is considered defaunated. We refer to this as a *boundary*, rather than an *endpoint*, because boundaries are crossable: A faunal community can both be depleted and rejuvenated. Defaunation, as we described above, is the point at which animals are absent. But totally defaunated systems are unlikely to exist, because even the most degraded pine monoculture includes hundreds of insect species. At the other extreme, in many places, insects are in a state of catastrophic population collapse (Goulson 2019) but are rarely taken into consideration in the defaunation literature. The forestry field deals with similar conundrums. For example, the FAO defines *forest* as an area having at least 10% forest cover, suggesting that an area is deforested when it has less than 10% forest cover (FAO 2000). This recognizes that the properties of a forest are lost well before every tree is removed and allows deforestation to be easily classified by measuring forest cover. To complement deforestation, the boundary at which a faunal community is deemed defaunated needs not be the complete absence of all animals. This would also facilitate the practical identification of defaunated ecosystems through emerging technologies (Pimm et al. 2015).

Defining the defaunation boundary is where we need to deviate from the deforestation concept. After all, *deforestation* only applies to forested ecosystems and is based on an observable and measurable physical change in them. By contrast, *defaunation* refers to all animal systems—terrestrial, aquatic, and marine. In addition, animals move, are arguably more difficult to monitor, and are not (usually) habitat, although they affect habitat. The defaunation boundary is therefore context specific and depends on conservation goals. For example, the management goals of an area might be to preserve biodiversity or to preserve functional groups that maintain ecosystem functioning. Even so, like the 90% threshold for deforestation, the defaunation boundary should be extreme—losing a high percentage of species or functional groups—to differentiate it from faunal degradation.

Because both faunal degradation and defaunation are context specific, they must be measured against a baseline, and the natural variation of that baseline must be understood (Thompson et al. 2013). A baseline is similar to a reference level in forest policy; it is the measure of animals in an area for a reference period, regardless of the index used. Once a baseline is defined, the degree of animal depletion can be measured (figure 1b). Moreover, it is also possible to quantify how close a system is to being defaunated. This raises the question of how baselines should be defined. Is the reference point the community composition before the Pleistocene mass extinctions, before people shaped terrestrial nature (Ellis et al. 2021), before the Industrial Revolution, or some desired reference (e.g., community composition before a specific human impact or date)? Recent studies have compared undisturbed (nonhunted) communities with disturbed (hunted) communities (Benítez-López et al. 2019), but this may not be appropriate in all situations. The baseline will depend on the management goal and will be context dependent, just as countries and projects set their reference levels for greenhouse gas emissions.

## Applying the framework

The initial step in applying this biodiversity framework to an area is to define the baseline on the basis of the management goal. The baseline for an area managed for species diversity might be determined by historical records and the earliest available survey data, whereas the baseline for maintaining ecosystem provisions, such as wild seafood, might be set to a relatively recent time before mechanized or commercial fishing. In any case, the baseline should be transparently determined and underpinned by ecology, not politics, such as establishing a baseline after an area has been defaunated for the purpose of avoiding investing resources in management. The definition of the baseline determines what needs to be monitored to quantify the magnitude of faunal degradation. Although the broadest definition of wildlife includes arthropods, it may not be feasible or necessary to monitor them. The final step is to delineate the boundary for defaunation where the loss of animals would provoke a state transition. In some ecosystems, the boundary might be high (e.g., 90% loss of animals), whereas in others, it might be lower, as the loss of keystone species induces a tropic cascade.

Our operational framework is well aligned with the goals and targets of the GBF. Its application can help guide conservation effort and policy as well as improve communication in science and to the lay public.

First, knowledge of whether a faunal community is defaunated, degraded, or converted can steer conservation planning, including deciding on priority locations and types of management intervention. GBF target 3 calls for the conservation and management of at least 30% of terrestrial, inland water, coastal, and marine areas by 2030. Making decisions around where to conserve requires information on the status of the animal community in an area. Expending effort and resources to conserve faunally degraded and converted novel ecosystems is likely to be more cost effective than investing in restoring defaunated ecosystems, although very little is known about the ability of different conservation interventions to deliver benefits at a given cost (Pienkowski et al. 2021). Similarly, whether an area is faunally degraded or defaunated will influence conservation interventions. Where all or most faunal species still exist (low faunal degradation), even at diminished abundances, passive measures—including the creation and management of protected areas, increasing connectivity between wildlife populations, and reduction of hunting—could replenish animal abundances. In defaunated or heavily degraded systems, active forms of species management might be required. These could include conservation translocation, including measures such as population restoration, reintroduction, assisted colonization, and ecological replacement (Seddon et al. 2014).

Second, quantifying the degree of faunal degradation can determine where on the faunal degradation curve a community or site lies (figure 1b). In the present article, we present a simple conceptual model based on species richness to measure degradation. Much more complex, rich statistics could be developed that incorporate animal abundances and species diversity, particularly as technologies for measuring animal abundances improve. As above, the degree of degradation might be factored into decisions regarding the prioritization of conservation sites or intervention types.

Forest initiatives developed through REDD+ often aspire to protect biodiversity as a cobenefit of protecting forests to reduce carbon emissions. Standard animal monitoring programs have even been proposed for REDD+ (Harrison et al. 2012). Measures of defaunation could be integrated into a policy framework whereby nations or projects are paid for reducing defaunation and faunal degradation. However, defaunation policy frameworks must avoid REDD+ pitfalls, such as failing to adequately recognize the rights of local and Indigenous peoples to natural resources. Many people, from Indigenous hunters (Nasi et al. 2011) to fisherman (Schindler et al. 2010, Halpern et al. 2012) depend on wildlife for their livelihoods. The GBF seems to recognize the conflict between strict protection and livelihood needs, simultaneously calling for a halt to the human-induced extinction of known endangered species (goal A) and the sustainable use of biodiversity for the benefit of present and future generations (goal B). Defaunation policies must be equitable and must contribute to both human livelihoods and ecosystem integrity.

Finally, loose or inaccurate use of language by scientists can have unintended consequences. Because of the use of terms such as *local extinction* rather than *extirpation*, extinction is no longer perceived as permanent in the eyes of the public (Smith-Patten et al. 2015). Likewise, defining *defaunation* as the loss of wildlife species or populations or declines in animals portrays a lack of exactness and definitude. It may not convey the sense of urgency necessary to garner support for conservation action in areas of most need. The loss or depletion of inconspicuous species while other charismatic species persist, for example, might seem academic or trivial to the public (although see Courchamp et al. 2018, suggesting that people are largely ignorant of the plight of charismatic species because of their profusion in our culture). Scientific warnings about a faunal community that later rebounds may further sentiment of scientists as alarmists. Indeed, the GBF calls for ensuring that biodiversity data are accessible to promote public education and informed governance and to strengthen communication and awareness raising (target 21).

There is potential for misuse of this framework that we need to warn against. Political leaders and decision-makers could misinterpret faunal degradation of 70%, for instance, as a nonpessimistic scenario that does not require management. Or they could cynically decide that any species loss short of defaunation does not merit investment. This would be a perversion of the framework that, quite to the contrary, is intended to alert scientists and policymakers of an impending problem before it is too late.

Defaunation and faunal degradation are topics of global concern because our society and economy are embedded in a natural system that is maintained by the activities of animals. We depend on wildlife for goods, such as food, medicine, and genetic resources, and services, including pollination, pest control, and water purification. This defaunation framework, aligned with the GBF, will advance the 2050 Vision for Biodiversity. Situating defaunation in an operational framework provides a standard means by which to conceptualize and quantify wildlife depletion. This enables the comparison of the state of faunal communities across sites. Most importantly, it also potentially provides a structure for finding solutions to mitigate or reverse the loss of wild animals.

## Supplementary Material

biad079_Supplemental_Table

## Author Biography

John Poulsen (john.poulsen@tnc.org) studies tropical ecology and conservation biology, particularly in forests in Central Africa, and is currently the global director for science capacity at The Nature Conservancy, in Boulder, Colorado, after working for 11 years as an associate professor of tropical ecology at Duke University, in Durham, North Carolina, in the United States. Vincent Maicher (vincent.maicher@duke.edu) was a postdoctoral scholar at Duke University and is now the CAFI Forest Research and Monitoring for The Nature Conservancy in Gabon. Halina Malinowski (halina.malinowski@duke.edu) and Camille DeSisto (camille.desisto@duke.edu) are PhD candidates, all affiliated with the Nicholas School of the Environment at Duke University.

## References

[bib1] Barnosky AD , et al. 2011. Has the Earth's sixth mass extinction arrived? Nature 471: 51–57 21368823 10.1038/nature09678

[bib2] Bar-On YM , Phillips R, Milo R. 2018. The biomass distribution on Earth. Proceedings of the National Academy of Sciences 115: 6506–6511. 10.1073/pnas.1711842115 PMC6016768 29784790

[bib3] Bayrak M , Marafa L. 2016. Ten years of REDD+: A critical review of the impact of REDD+ on forest-dependent communities. Sustainability 8: 620.

[bib4] Benítez-López A , Alkemade R, Schipper AM, Ingram DJ, Verweij PA, Eikelboom JAJ, Huijbregts MAJ. 2017. The impact of hunting on tropical mammal and bird populations. Science 356: 180–183. 28408600 10.1126/science.aaj1891

[bib5] Benítez-López A , Santini L, Schipper AM, Busana M, Huijbregts MAJ. 2019. Intact but empty forests? Patterns of hunting-induced mammal defaunation in the tropics. PLOS Biology 17: e3000247. 31086365 10.1371/journal.pbio.3000247 PMC6516652

[bib7] Bogoni JA , Pires JSR, Graipel ME, Peroni N, Peres CA. 2018. Wish you were here: How defaunated is the Atlantic Forest biome of its medium- to large-bodied mammal fauna? PLOS ONE 13: e0204515. 30252909 10.1371/journal.pone.0204515 PMC6155554

[bib6] Bogoni JA , Peres CA, Ferraz KM. 2020. Effects of mammal defaunation on natural ecosystem services and human well-being throughout the entire neotropical realm. Ecosystem Services 45: 101173.

[bib8] Borrvall C , Ebenman B, Tomas Jonsson TJ. 2000. Biodiversity lessens the risk of cascading extinction in model food webs. Ecology Letters 3: 131–136.

[bib9] Brook B , Sodhi N, Bradshaw C. 2008. Synergies among extinction drivers under global change. Trends in Ecology and Evolution 23: 453–460. 18582986 10.1016/j.tree.2008.03.011

[bib10] Ceballos G , Ehrlich PR, Barnosky AD, García A, Pringle RM, Palmer TM. 2015. Accelerated modern human-induced species losses: Entering the sixth mass extinction. Science Advances 1: e1400253. 26601195 10.1126/sciadv.1400253 PMC4640606

[bib11] Ceballos G , Ehrlich PR, Raven PH. 2020. Vertebrates on the brink as indicators of biological annihilation and the sixth mass extinction. Proceedings of the National Academy of Sciences 117: 13596–13602. 10.1073/pnas.1922686117 PMC7306750 32482862

[bib12] Cerini F , Childs DZ, Clements CF. 2023. A predictive timeline of wildlife population collapse. Nature Ecology and Evolution 7: 320–331. 36702859 10.1038/s41559-023-01985-2

[bib13] Chaudhary V , Oli MK. 2020. A critical appraisal of population viability analysis. Conservation Biology 34: 26–40. 31435956 10.1111/cobi.13414

[bib14] Courchamp F , Jaric I, Albert C, Meinard Y, Ripple WJ, Chapron G. 2018. The paradoxical extinction of the most charismatic animals. PLOS Biology 16: e2003997. 29649205 10.1371/journal.pbio.2003997 PMC5896884

[bib15] Cowie RH , Bouchet P, Fontaine B. 2022. The sixth mass extinction: Fact, fiction, or speculation? Biological Reviews 97: 640–663. 35014169 10.1111/brv.12816 PMC9786292

[bib16] Dirzo R , Miranda A. 1991. Altered patterns of herbivory and diversity in the forest understory: A case study of the possible consequences of contemporary defaunation. Pages 273–287 in Price PW, Lewinsohn TM, Fernandes GW, Benson WW, eds. Plant–Animal Interactions: Evolutionary Ecology in Tropical and Temperate Regions. Wiley-Interscience.

[bib17] Dirzo R , Young HS, Galetti M, Ceballos G, Isaac NJB, Collen B. 2014. Defaunation in the Anthropocene. Science 401: 401–406. 10.1126/science.1251817 25061202

[bib18] Ellis EC , et al. 2021. People have shaped most of terrestrial nature for late last 12,000 years. Proceedings of the National Academy of Sciences 188: e2023483118. 10.1073/pnas.2023483118 PMC8092386 33875599

[bib19] Estes JA , et al. 2011. Trophic downgrading of Planet Earth. Science 333: 301–306. 21764740 10.1126/science.1205106

[bib20] [FAO] Food and Agriculture Organization of the United Nations . 2000. FRA 2000: On Definitions of Forest and Forest Cover Change. FAO. 6086142

[bib21] Finderup Nielsen T , Sand-Jensen K, Dornelas M, Bruun HH. 2019. More is less: Net gain in species richness, but biotic homogenization over 140 years. Ecology Letters 22: 1650–1657. 31364805 10.1111/ele.13361

[bib22] Fraser D , et al. 2022. Late quaternary biotic homogenization of North American mammalian faunas. Nature Communications 13: 3940. 10.1038/s41467-022-31595-8 PMC9270452 35803946

[bib23] Fuzessy LF , Beníıtez-López A, Slade EM, Bufalo FS, Magro-de-Souza GC, Pereira LA, Culot L. 2021. Identifying the anthropogenic drivers of declines in tropical dung beetle communities and functions. Biological Conservation 256: 109063.

[bib24] Giacomini HC , Galetti M. 2013. An index for defaunation. Biological Conservation 163: 33–41.

[bib25] Goetz SJ , Hansen M, Houghton RA, Walker W, Laporte N, Busch J. 2015. Measurement and monitoring needs, capabilities and potential for addressing reduced emissions from deforestation and forest degradation under REDD+. Environmental Research Letters 10: 123001.

[bib26] Goulson D . 2019. The insect apocalypse, and why it matters. Current Biology 29: R967–R971. 31593678 10.1016/j.cub.2019.06.069

[bib27] Hallmann CA , et al. 2017. More than 75 percent decline over 27 years in total flying insect biomass in protected areas. PLOS ONE 12: e0185809. 29045418 10.1371/journal.pone.0185809 PMC5646769

[bib28] Halpern BS , et al. 2012. An index to assess the health and benefits of the global ocean. Nature 488: 615–620. 22895186 10.1038/nature11397

[bib29] Harrison ME , Boonman A, Cheyne SM, Husson SJ, Marchant NC, Struebig MJ. 2012. Biodiversity monitoring protocols for REDD+: Can a one-size-fits-all approach really work? Tropical Conservation Science 5: 1–11.

[bib30] Hosonuma N , Herold M, De Sy V, De Fries RS, Brockhaus M, Verchot L, Angelsen A, Romijn E. 2012. An assessment of deforestation and forest degradation drivers in developing countries. Environmental Research Letters 7: 044009.

[bib31] McCauley DJ , Pinsky ML, Palumbi SR, Estes JA, Joyce FH, Warner RR. 2015. Marin defaunation: Animal loss in the global ocean. Science 347: 1255641. 25593191 10.1126/science.1255641

[bib32] Merriam-Webster . 2023. Deforestation. Merriam-Webster. www. merriam-webster.com/dictionary/deforestation.

[bib33] Morse NB , Pellissier PA, Cianciola EN, Brereton RL, Sullivan MM, Shonka NK, Wheeler TB, McDowell WH. 2014. Novel ecosystems in the Anthropocene: A revision of the novel ecosystem concept for pragmatic applications. Ecology and Society 19: 12.

[bib34] Myers RA , Baum JK, Shepherd TD, Powers SP, Peterson CH. 2007. Cascading effects of the loss of apex predatory sharks from a coastal ocean. Science 315: 1846–1850. 17395829 10.1126/science.1138657

[bib35] Nasi R , Taber A, Van Vliet N. 2011. Empty forests, empty stomachs? Bushmeat and livelihoods in the Congo and Amazon Basins. International Forestry Review 13: 355–368.

[bib36] Pacoureau N , et al. 2021. Half a century of global decline in oceanic sharks and rays. Nature 589: 567–571. 33505035 10.1038/s41586-020-03173-9

[bib37] Pereira HM , et al. 2010. Scenarios for global biodiversity in the 21st century. Science 330: 1496–1501. 20978282 10.1126/science.1196624

[bib38] Pienkowski T , Cook C, Verma M, Carrasco LR. 2021. Conservation cost-effectiveness: A review of the evidence base. Conservation Science and Practice 3: e357.

[bib40] Pimm SL , Jenkins CN, Abell R, Brooks TM, Gittleman JL, Joppa LN, Raven PH, Roberts CM, Sexton JO. 2014. The biodiversity of species and their rates of extinction, distribution, and protection. Science 344: 1246752. 24876501 10.1126/science.1246752

[bib39] Pimm SL , Alibhai S, Bergl R, Dehgan A, Giri C, Jewell Z, Joppa L, Kays R, Loarie S. 2015. Emerging technologies to conserve biodiversity. Trends in Ecology and Evolution 30: 685–696. 26437636 10.1016/j.tree.2015.08.008

[bib41] Plumptre AJ , et al. 2021. Where might we find ecologically intact communities? Frontiers in Forests and Global Change 4: 626635.

[bib42] Poulsen JR , Clark CJ, Bolker BM. 2011. Decoupling the effects of logging and hunting on an afrotropical animal community. Ecological Applications 21: 1819–1836. 21830721 10.1890/10-1083.1

[bib43] Poulsen JR , Clark CJ, Palmer TM. 2013. Ecological erosion of an afrotropical forest and potential consequences for tree recruitment and forest biomass. Biological Conservation 163: 122–130.

[bib44] Putz FE , Redford KH. 2010. The importance of defining “forest”: Tropical forest degradation, deforestation, long-term phase shifts, and further transitions. Biotropica 42: 10–20.

[bib45] Redford KH . 1992. The Empty Forest. Bioscience 42: 412–422.

[bib46] Schindler DE , Hilborn R, Chasco B, Boatright CP, Quinn TP, Rogers LA, Webster MS. 2010. Population diversity and the portfolio effect in an exploited species. Nature 465: 609–612. 20520713 10.1038/nature09060

[bib47] Seddon PJ , Griffiths CJ, Soorae PS, Armstrong DP. 2014. Reversing defaunation: Restoring species in a changing world. Science 345: 406–412. 25061203 10.1126/science.1251818

[bib48] Smith FA , Elliott Smith RE, Lyons SK, Payne JL. 2018. Body size downgrading of mammals over the late quaternary. Science 360: 310–313. 29674591 10.1126/science.aao5987

[bib49] Smith-Patten BD , Bridge ES, Crawford PH, Hough DJ, Kelly JF, Patten MA. 2015. Is extinction forever? Public Understanding of Science 24: 481–495. 25711479 10.1177/0963662515571489 PMC4404403

[bib50] Terborgh J , et al. 2001. Ecological meltdown in predator-free forest fragments. Science 294: 1923–1926. 11729317 10.1126/science.1064397

[bib51] Thompson ID , Guariguata MR, Okabe K, Bahamondez C, Nasi R, Heymell V, Sabogal C. 2013. An operational framework for defining and monitoring forest degradation. Ecology and Society 18: 20.

[bib52] Tropek R , Sedláček O, Beck J, Keil P, Musilová Z, Šímová I, Storch D. 2014. Comment on “High-resolution global maps of 21st-century forest cover change.” Science 344: 981–981. 10.1126/science.1248753 24876487

[bib53] [UNFCCC] United Nations Framework Convention on Climate Change . 2001. The Marrakesh Accords and the Marrakesh Declaration. UNFCCC. https://unfccc.int/cop7/documents/accords_draft.pdf.

[bib54] Vasquez-Grandon A , Donoso PJ, Gerding V. 2018. Forest degradation: When is a Forest degraded? Forests 9: 726.

[bib55] Williams PJ , Ong RC, Brodie JF, Luskin MS. 2021. Fungi and insects compensate for lost vertebrate seed predation in an experimentally defaunated tropical forest. Nature Communications 12: 1650. 10.1038/s41467-021-21978-8 PMC7955059 33712621

[bib56] [WWF] World Wildlife Fund . 2020. Living Planet Report 2020: Bending the Curve of Biodiversity Loss. WWF.

[bib57] Young HS , McCauley DJ, Galetti M, Dirzo R. 2016. Patterns, causes, and consequences of Anthropocene defaunation. Annual Review of Ecology, Evolution, and Systematics 47: 333–358.

